# Linezolid in Combination With Azoles Induced Synergistic Effects Against *Candida albicans* and Protected *Galleria mellonella* Against Experimental Candidiasis

**DOI:** 10.3389/fmicb.2018.03142

**Published:** 2019-01-31

**Authors:** Mengjiao Lu, Xinmei Yang, Cuixiang Yu, Ying Gong, Lei Yuan, Lina Hao, Shujuan Sun

**Affiliations:** ^1^School of Pharmaceutical Sciences, Shandong University, Ji’nan, China; ^2^Department of Pharmacy, Baodi People’s Hospital, Tianjin, China; ^3^Department of Pharmacy, Qianfoshan Hospital Affiliated to Shandong University, Ji’nan, China; ^4^Department of Respiration Medicine, Qianfoshan Hospital Affiliated to Shandong University, Ji’nan, China; ^5^Qilu Children’s Hospital of Shandong University, Ji’nan, China

**Keywords:** linezolid, azoles, synergism, *Candida albicans*, *Galleria mellonella* model

## Abstract

The incidence of resistant *Candida* isolates has increased continuously in recent decades, especially *Candida albicans*. To overcome this resistance, research on antifungal sensitizers has attracted considerable attention. Linezolid was found to inhibit the growth of *Pythium insidiosum* and synergize with amphotericin B against *Cryptococcus neoformans*. The objective of this study was to determine the interactions of linezolid and azoles against *C. albicans in vitro* and *in vivo*. *In vitro*, linezolid combined with azoles induced synergistic effects not only against some susceptible *C. albicans* isolates, but also against all tested resistant *C. albicans* isolates. For all resistant isolates, exposure to the combination of linezolid with azoles induced a significant decrease in the minimum inhibitory concentrations (MIC) of azoles, from >512 to 0.5–1 μg/mL for fluconazole, from >16 to 0.25–1 μg/mL for itraconazole, and from >16 to 0.03–0.25 μg/mL for voriconazole. Additionally, linezolid synergized with fluconazole against biofilms that were preformed for ≤ 12 h from both susceptible and resistant *C. albicans*, and the sessile MIC of fluconazole decreased from >1024 to 1–4 μg/mL. *In vivo*, linezolid plus azoles prolonged the survival rate of infected *Galleria mellonella* larvae twofold compared with the azole monotherapy group, significantly decreased the fungal burden of the infected larvae, and reduced the damage of resistant *C. albicans* to the larval tissue. These findings will contribute to antifungal agent discovery and new approaches for the treatment of candidiasis caused by *C. albicans*.

## Introduction

Invasive fungal infections, especially the blood infection caused by *Candida* species, have become a major cause of morbidity and mortality in recent decades (Kriengkauykiat et al., 2011; Kullberg and Arendrup, 2015). *Candida* species are fungal organisms known to affect immunocompromised patients and are known to cause superficial infection of skin, mouth, or mucous membranes as well as invasive infections such as candidemia and biofilm-related infection (Sardi et al., 2013; Polesello et al., 2017). In *Candida* infections, *Candida albicans* is the most isolated strain. Data from the Prospective Antifungal Therapy Alliance registry showed that among the 7526 fungi isolated from 6807 invasive fungal infections, the highest isolation rate was for *Candida* (*n* = 5526, 73.4%), and *C. albicans* accounted for 47.8% of its isolation rate (Azie et al., 2012). Azoles such as fluconazole (FLC), itraconazole (ITZ) and voriconazole (VRC) possess great efficacy and lower toxicity, and there is an extensive use of azoles in clinical practice to prevent and treat candidiasis. However, along with the increase of infection and extensive application of azoles, drug-resistant strains have frequently emerged (Whaley et al., 2016; Berkow and Lockhart, 2017; Perlin et al., 2017). For example, a twofold increase in the rate of resistance to azoles among *C. albicans* isolates was observed from 2010 (0.4 for FLC and 0.7% for VRC) to 2014 (0.8 for FLC and 1.5% for VRC) in China (Xiao et al., 2018). In addition, a clearly higher FLC resistance rate of *C. albicans* was observed in some cities of China as well as other areas, such as Nanchang (4.3%) (Ying et al., 2015), Hefei (4.5%) (Zhang et al., 2015), two US cities (2.3%) (Lockhart et al., 2012), Australia (3.46%) (Espinel-Ingroff et al., 2014) and Medellín (7.3%) (Maldonado et al., 2014). Therefore, research on antifungal sensitizers to overcome fungal resistance has attracted considerable attention (Rodrigues et al., 2016).

Linezolid (LZD) is the first oxazolidinone antibiotic and possesses good activity against gram-positive pathogens, including multidrug-resistant pathogens, methicillin-resistant *Staphylococcus aureus* (MRSA), and vancomycin-resistant *Enterococci* (VRE; Mendes et al., 2014; Zahedi Bialvaei et al., 2017). It was approved by the FDA in the year 2000 for the treatment of multidrug resistant gram-positive bacterial infections. Recently, extensive efforts have been devoted to finding new antimicrobial activities of LZD, such as antituberculotic (Lee et al., 2015; Sotgiu et al., 2015), antileishmanial (Limoncu et al., 2013), and antifungal effects (Kamal et al., 2013; Loreto et al., 2014; Rossato et al., 2015). Studies on the antifungal effects of LZD found that LZD exerts an inhibitory effect against *Pythium insidiosum* with a minimum inhibitory concentration (MIC) ≤ 16 μg/mL (Loreto et al., 2014), and multiple LZD-like oxazolidino-sulfonamides possessed antifungal activities against *C. albicans*, with a MIC value of 4.0 μg/mL (Kamal et al., 2013). Furthermore, LZD was found to work synergistically with amphotericin B against clinical isolates of *Cryptococcus neoformans* (Rossato et al., 2015). However, there is no study on the combination of LZD and azoles against *C. albicans*. Therefore, this study systematically evaluated the combined effects of LZD with FLC against *C. albicans* both *in vitro* and *in vivo*.

Although the *in vivo* studies in microbiology are commonly performed using mammals as an infection model, invertebrate models have gained considerable attention as a viable alternative to traditional mammalian models of infection. The larvae of the wax moth, *Galleria mellonella*, have been used as an alternative infection model since the 1980s and have been increasingly used to study multiple gram-positive bacteria, gram-negative bacteria, and several pathogenic fungi and viruses (Champion et al., 2016). Compared with the mammalian models, *G. mellonella* larvae possess the advantage of being cheaper and easier to maintain and handle (Champion et al., 2016; Tsai et al., 2016). In addition, as an invertebrate, the *G. mellonella* larva infection model does not require ethical approval and could provide a rapid evaluation of pathogen virulence or efficacy of antimicrobial treatment because of their short life span (Kwadha et al., 2017). It is worth mentioning that *G. mellonella* larvae can be maintained at temperatures between 15 and 37°C, suitable for studies at human body temperature (Desalermos et al., 2012). Of note, *G. mellonella* larvae have both a cellular and humoral immune response to infection, similar to mammals (Wojda, 2017). These advantages make *G. mellonella* larvae an attractive host for studying pathogens and antimicrobial agents.

In the present study, the microdilution method was conducted to evaluate the *in vitro* antifungal activity of LZD alone or in combination with azoles, and the antibiofilm effects of LZD combined with FLC were determined by the XTT assay. In addition, the interactions of drug combinations *in vivo* were evaluated by establishing the *G. mellonella* larva infection model and assaying the impact of LZD and azoles used alone and in combination on the survival rate and fungal burden as well as the histological section of the larvae.

## Materials and Methods

### Strains and Media

The strains used in this study are listed in Table 1. CA103, CA632, and CA20003 were kindly provided by Professor Changzhong Wang (School of Integrated Traditional and Western Medicine, Anhui University of Traditional Chinese Medicine, Hefei, China), and others were isolated from the clinical laboratory of Shandong Provincial Qianfoshan Hospital, Jinan, China. *C. albicans* ATCC10231, a quality control for determining MICs of drugs, was kindly donated by the Institute of Pharmacology, School of Pharmacy, Shandong University, Ji’nan, Shandong Province, China. All strains were stored as frozen stocks of isolates in the Sabouraud dextrose broth (SDB) at –80°C and were subcultured on Sabouraud dextrose agar (SDA) for 24 h at 35°C at least twice before the experiment. For the *in vivo* experiment, *G. mellonella* larvae similar in size (ca. 0.25 g) and absent of gray markings in the final instar were chosen to be used.

**Table 1:** *In vitro* interactions of LZD with azoles against *Candida albicans*.

Strains^a^	MIC_80_ (μg/mL)^b^				FICI^b^	IN^c^
	Alone		Combined			
	Azoles	LZD	Azoles	LZD		
***FLC***						
CA4	0.5	>512	0.25	32	0.56	IND
CA8	2	>512	0.5	32	0.31	SYN
CA14	2	>512	1	128	0.75	IND
CA17	0.5	>512	0.25	8	0.52	IND
CA19	0.5	>512	0.5	8	1.02	IND
CA23	1	>512	0.25	32	0.31	SYN
CA10	>512	>512	1	32	0.06	SYN
CA16	>512	>512	1	32	0.06	SYN
CA103	>512	>512	0.5	16	0.03	SYN
CA137	>512	>512	1	16	0.03	SYN
CA632	>512	>512	1	32	0.06	SYN
CA20003	>512	>512	1	32	0.06	SYN
***ITZ***						
CA4	0.25	>512	0.25	8	1.02	IND
CA8	0.5	>512	0.25	8	0.52	IND
CA14	1	>512	0.5	32	0.56	IND
CA17	1	>512	0.5	16	0.53	IND
CA19	0.125	>512	0.125	16	1.03	IND
CA23	2	>512	0.5	32	0.31	SYN
CA10	>16	>512	1	32	0.13	SYN
CA16	>16	>512	0.25	32	0.08	SYN
CA103	>16	>512	1	64	0.19	SYN
CA137	>16	>512	0.5	32	0.09	SYN
CA632	>16	>512	0.5	32	0.09	SYN
CA20003	>16	>512	0.5	32	0.09	SYN
***VRC***						
CA4	0.03	>512	0.008	16	0.29	SYN
CA8	0.5	>512	0.06	32	0.19	SYN
CA14	0.5	>512	0.03	16	0.09	SYN
CA17	0.125	>512	0.03	16	0.28	SYN
CA19	0.03	>512	0.02	32	0.56	IND
CA23	0.13	>512	0.03	32	0.31	SYN
CA10	>16	>512	0.25	32	0.08	SYN
CA16	>16	>512	0.06	32	0.07	SYN
CA103	>16	>512	0.06	32	0.07	SYN
CA137	>16	>512	0.06	32	0.07	SYN
CA632	>16	>512	0.06	32	0.07	SYN
CA20003	>16	>512	0.03	64	0.13	SYN

*^a^CA, Candida albicans; ^b^MIC_80,_ minimum inhibitory concentration of drug that inhibited fungal growth by 80% compared with the growth control; FICI, fractional inhibitory concentration index; MIC_80_ values and FICIs are the median of three independent experiments; and ^c^IN, interpretation; SYN, synergism; IND, indifference.*

### Antimicrobial Agents

Antimicrobial agents (FLC, LZD, ITZ, VRC, and ampicillin) were purchased from Dalian Meilun Biotech Co., Ltd., China. Following the manufacturer’s instructions, stock solutions of FLC, LZD, VRC, and ITZ were dissolved with dimethylsulfoxide (DMSO), and ampicillin was prepared in sterile distilled water. Stock solutions were all sterilized using 0.22 micro filters, aliquoted and stored at –20°C until use.

### Antifungal Activities of LZD and Azoles

The antifungal activities of all tested drugs against *C. albicans* were determined using the broth microdilution method according to the Clinical and Laboratory Standards Institute standard M27-A3 document (CLSI, M27-A3). The test was performed with the yeast (2.5 × 10^3^ CFU/mL) in RPMI-1640 medium (PH 7.0) buffered with MOPS [morpholino (propanesulfonic acid)] in 96-well microtiter plates. Drugs at final concentrations of 1–512 μg/mL for LZD, 0.125–512 μg/mL for FLC, and 0.006–16 for ITZ and VRC were added to the wells. The wells containing RPMI 1640 medium acted as negative controls, and a drug-free well was set as the growth control. After 24 h of incubation at 35°C, the MICs were determined by both visual reading and measuring the optical density (OD) with a microplate reader at 492 nm. The MICs were defined as, compared with that of the drug-free control, the lowest concentration of drug that still enable 80% fungal growth inhibition.

### Checkerboard Methods

The interactions of LZD with azoles against *C. albicans* were assessed using the broth microdilution checkerboard method according to the CLSI guidelines for yeast (document M27-A3). Drugs were serially diluted in RPMI-1640 medium, and the final concentrations were 4–256 μg/mL for LZD, 0.25–128 μg/mL for FLC and 0.0625–16 μg/mL for VRC and ITZ. The cell suspension was subsequently added to each well at a final concentration of 2 × 10^3^ CFU/mL. Plates were incubated for 24 h or 48 h at 35°C and the growth in each well was then quantified by both visual observation and a microplate reader in the same manner as the susceptibility testing.

To evaluate the mode and intensity of the drug interactions, the obtained data were analyzed using two models: the fractional inhibitory concentration index (FICI) model based on the Loewe additivity theory and Δ*E* model based on the Bliss independence (BI) theory. The FICI was calculated by the following equation: FICI = FIC_A_+FIC_B_ = MIC_(A–combo)_/MIC_(A–alone)_ + MIC_(B–combo)_/MIC_(B–alone)_ and interpreted as synergistic when FICI ≤ 0.5, antagonistic when FICI ≥ 4 and indifferent when FIC < 0.5–4. The Δ*E* model was described by the following equation: Δ*E* = *E*_A_ ×*E*_B_ – *E*_measured_, where *E*_A_ and *E*_B_ are the experimental percentages of growth when drugs act alone, and *E*_measured_ is the measured percentage of growth with the theoretical combination of drugs A and B. When the mean Δ*E* as well as its 95% confidence interval is positive, significant synergy is claimed. When the mean Δ*E* as well as its 95% confidence interval is negative, significant antagonism is claimed. In any other case, the conclusion was Bliss independence. To summarize the entire interaction, the sum percentages of all significant synergistic (ΣSYN) or antagonistic (ΣANT) interactions were calculated. Interactions with 200% were considered strong, those with 100–200% were considered moderate, and those with <100% were considered weak.

### Antibiofilm Activity Testing

The interactions of LZD with FLC against biofilms of *C. albicans* were assessed in 96-well plates as previously described with some modifications (Zhong et al., 2017). Before this test, biofilm production of the isolates used in this study were evaluated using the method described by Shin et al. (2002) with some modifications, and all isolates except CA17 and CA20003 were found to be high biofilm producers (Supplementary Table S1). Thus, three high biofilm-producing strains (CA4, CA8, and CA10) were selected to determine the antibiofilm activity. Briefly, aliquots of 200-μL yeast suspension (2.5 × 10^3^ CFU/mL) were added to a 96-well plate and the plates were incubated over four time intervals (4, 8, 12, and 24 h) at 35°C to preform the biofilms at different stages of maturation. Then, the preformed biofilms were washed with sterile phosphate-buffered saline (PBS) three times, and drugs were added to the biofilm-coated wells at final concentrations of 2–1024 μg/mL for FLC and 16–1024 μg/mL for LZD. Following a further 24 h of incubation at 35°C, an XTT reduction assay was performed to examine the metabolic activity of the biofilms. Colorimetric changes in the XTT reduction were measured with a microplate reader at 492 nm. The sessile minimum inhibitory concentration (sMIC) was defined as, compared with that of the drug-free control, the lowest concentration of drug that would lead to an 80% inhibition of biofilm metabolic activity.

### *Galleria mellonella* Infection Model

#### *G. mellonella* Survival Assay

For the primary determination of the *in vivo* combined effects of LZD and FLC, a *G. mellonella* survival assay was performed according to a previously described methodology (Gu et al., 2016, 2017). In brief, seven groups of randomly chosen larvae (20 for each group) were infected with 10 μL *C. albicans* (CA10) inoculums (5 × 10^8^ CFU/mL) using a 50-μL microsyringe *via* the last left proleg of the larvae. Three hours after the infection, larvae in each group were injected *via* the last right proleg of the larvae with 10 μL of sterile PBS, FLC (160 μg/mL), ITZ (40 μg/mL), VRC (40 μg/mL), LZD (200 μg/mL), LZD (200 μg/mL) plus FLC (160 μg/mL), LZD (200 μg/mL) plus ITZ (40 μg/mL), and LZD (200 μg/mL) plus VRC (40 μg/mL), respectively. Another group of randomly chosen larvae only injected with 10 μL sterile PBS plus 10 μL sterile PBS served as the blank control group. Larvae were then incubated at 35°C in plastic containers and monitored daily for survival for 4 days. A larva was considered dead when it displayed no response to touch. Larvae were cleaned by an alcohol swab prior to injection and were placed in the dark at 35°C during the experiments.

#### Fungal Burden Analysis

To evaluate the effect of the combination of LZD and FLC on the fungal burden of larvae infected with *C. albicans* (CA10), the colony count method was used. Four groups of randomly selected larvae (20 for each group) were injected with the yeast inoculums and drugs as described above and incubated at 35°C for 4 days. During the incubation, three larvae from each group were randomly taken daily, washed with 70% ethanol and homogenized in 3 mL sterile PBS-ampicillin. Then, the homogenate of each group was serially diluted 10-fold with sterile PBS-ampicillin, and a 10-μL suspension was placed on YPD agar. Colony counts were performed after incubation for 24–48 h at 35°C.

#### Histological Study

A histological study was performed to observe the effect of the combination of LZD and FLC on the tissue of *G. mellonella* larvae infected with *C. albicans* (CA10). Four groups of randomly selected larvae were injected with yeast inoculums and drugs as described above and another group of larvae were untreated with yeast and drugs. Two larvae from each group were taken after two-day injection, washed with ethanol and cut into histological sections (20 μm). Sections were then stained with periodic acid Schiff (PAS) reagent and observed under a fluorescence microscope with 4.2 × magnification.

### Statistical Analysis

All experiments were performed three times on different days. Graphs were created and statistical analyses were performed with GraphPad Prisma 5 and SPSS Statistics V17.0. Survival curves and differences were analyzed using the Kaplan–Meier method and the log-rank test. Data of fungal burden were statistically analyzed using Student’s *t*-test. *P* < 0.05 was considered significant.

## Results

### LZD in Combination With Azoles Induced Synergistic Effects Against *C. albicans*

The MICs of azoles and LZD were assessed against twelve *C. albicans* isolates, and data are shown in Table 1. The first six *C. albicans* isolates were FLC-susceptible with the MICs of FLC ranging from 0.5 to 2 μg/mL, and the other six *C. albicans* isolates were FLC-resistant with all MICs > 512 μg/mL. In addition, the MICs of LZD against the tested strains were all >512 μg/mL, demonstrating a very limited intrinsic antifungal activity.

For the interactions of LZD with azoles against *C. albicans*, when LZD was combined with azoles against the susceptible isolates, the synergism was observed only in some of the six tested isolates with FICI values in the range of 0.09–0.31: CA8 and CA23 for the combination of LZD and FLC, CA23 for the combination of LZD and ITZ, and other isolates other than CA19 for the combination of LZD and VRC. Indifference was observed in the remaining isolates, and no antagonism was observed in the combination of LZD and azoles against all susceptible strains.

**FIGURE 1:**
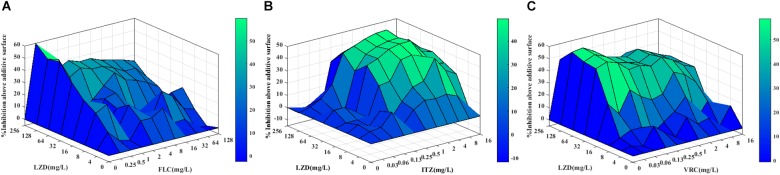
Three-dimensional model of LZD combined with azoles against CA10 *in vitro*. **(A–C)** The three-dimensional model of LZD with FLC, ITZ and VRC, respectively. The Δ*E* values are depicted on the *z*-axis, and the peaks above the 0 plane indicate synergistic combinations, whereas the peaks below the 0 plane indicate antagonistic combinations.

Notably, when used in combination with azoles against FLC-resistant *C. albicans*, LZD in a dose of 16–32 μg/mL significantly decreased the MICs of azoles from >512 to 0.5–1 μg/mL for FLC, from >16 to 0.25–1 μg/mL for ITZ, and from >16 to 0.03–0.25 μg/mL for VRC, showing that LZD could significantly increase the sensitivity of resistant *C. albicans* to azoles. The FICI values were 0.03–0.06 for the combination of LZD and FLC, 0.08–0.19 for the combination of LZD and ITZ, and 0.07–0.13 for the combination of LZD and VRC. All FICI values were substantially less than 0.5, showing strong synergistic effects between LZD and azoles against resistant *C. albicans*. Additionally, the synergism was demonstrated by the Δ*E* model (Figure 1), with most of the Δ*E* values above the 0 plane. These observations indicated that LZD combined with azoles synergistically inhibited the growth of *C. albicans*, especially the growth of resistant *C. albicans*, and LZD might be a candidate for combination with azoles against drug-resistant *C. albicans*.

### LZD Synergized With FLC Against Different Stages of *C. albicans* Biofilms

Three high biofilm-producing strains (CA4, CA8, and CA10) were used to test the interactions of LZD with FLC against preformed biofilms, and the results were interpreted by a FICI model as mentioned above (Table 2). The results showed that for the biofilms preformed for ≤ 12 h of these three isolates, LZD decreased the sMIC_80_ of FLC from >1024 to 1–4 μg/mL with the FICI values ranging from 0.03 to 0.25. The FICI values were all substantially less than 0.5, showing a strong synergism between LZD and FLC. For the biofilms preformed over 24 h, the sMIC_80_ of FLC showed almost no change when combined with LZD compared with FLC alone, and the FICI values were 2, indicating indifferent interactions between LZD and FLC. These data indicated that LZD could work synergistically with FLC against biofilms formed at an early stage but not more mature biofilms.

**Table 2:** *In vitro* interactions of LZD with FLC against *C. albicans* biofilms.

Isolates^a^	Time (h)^b^	sMIC_80_ of drugs (μg/ml)^c^				FICI^c^	IN^d^
		Alone		Combined			
				
		FLC	LZD	FLC	LZD		
CA4	4	>1024	>1024	1	32	0.03	SYN
	8	>1024	>1024	2	64	0.06	SYN
	12	>1024	>1024	2	128	0.13	SYN
	24	>1024	>1024	>1024	>1024	2	IND
CA8	4	>1024	>1024	2	32	0.03	SYN
	8	>1024	>1024	2	64	0.06	SYN
	12	>1024	>1024	4	128	0.13	SYN
	24	>1024	>1024	>1024	>1024	2	IND
CA10	4	>1024	>1024	2	64	0.06	SYN
	8	>1024	>1024	2	128	0.13	SYN
	12	>1024	>1024	2	256	0.25	SYN
	24	>1024	>1024	>1024	>1024	2	IND

*^a^CA, Candida albicans; ^b^Incubation period of pre-formed biofilm; ^c^sMIC_80,_ minimum inhibitory concentration of drug that inhibited fungal growth by 80% compared with the growth control; FICI, fractional inhibitory concentration index; MIC_80_ values and FICIs are the median of three independent experiments; and ^d^IN, interpretation; SYN, synergism; IND, indifference.*

### LZD Protected *G. mellonella* Larvae Against Experimental Candidiasis

#### Survival Assay

**FIGURE 2:**
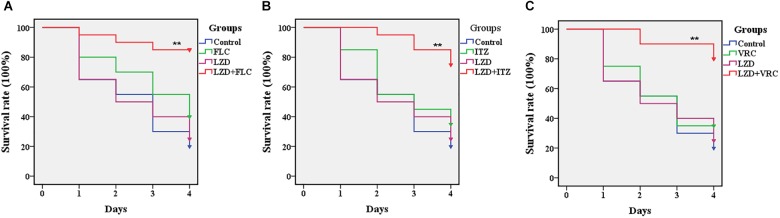
Survival rate of infected *Galleria mellonella* larvae treated with different drugs. Panels **(A–C)** are the survival rates of infected *Galleria mellonella* treated with LZD + FLC, LZD + ITZ, and LZD + VRC, respectively. CA10 (5 × 10^8^ CFU/mL) was used to infect the *G. mellonella* larvae (20 larvae for each group). After infection, the larvae were treated with sterile PBS, FLC (160 μg/mL), ITZ (40 μg/mL), VRC (40 μg/mL), LZD (200 μg/mL), LZD (200 μg/mL) plus FLC (160 μg/mL), LZD (200 μg/mL) plus ITZ (40 μg/mL), and LZD (200 μg/mL) plus VRC (40 μg/mL), respectively. The data came from the means of three independent experiments and the log-rank test was performed. Compared with the FLC-treated group, ^∗^*P* < 0.05, ^∗∗^*P* < 0.01.

In this study, *G. mellonella* larvae infected with CA10 were used to evaluate the *in vivo* interactions of drug combinations, and the survival assay was performed to primarily evaluate the *in vivo* interactions. The data of the survival assay showed that 20% of the infected larvae in the control group survived during the 4-day infection, and the survival rate of the larvae treated with LZD alone was 25%, not significantly different from that of the control group (*P* < 0.05) (Figure 2). With the monotherapy of azoles, the survival rates of the larvae were 40% for FLC and 35% for ITZ and VRC, slightly higher than that of the control group, indicating weak effects of azole monotherapy on the infected larvae. Of note, LZD plus azoles protected the larvae from *C. albicans* infection and resulted in 75–85% of the larvae surviving until the end of the observation period. More specifically, the survival rate of the combination groups was 85% for LZD + FLC, 75% for LZD + ITZ and 80% for LZD + VRC, showing significantly increased survival rates of larvae using the combination of LZD and azoles (*P* < 0.01).

#### Fungal Burden Analysis

**FIGURE 3:**
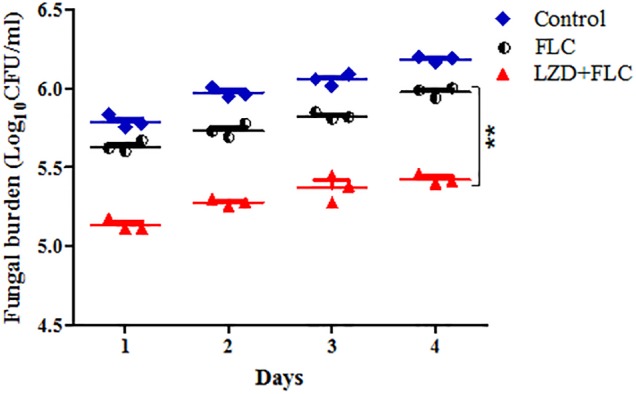
Fungal burden of infected *G. mellonella* larvae treated with different drugs. CA10 (5 × 10^8^ CFU/mL) was used to infect the *G. mellonella* larvae (20 larvae for each group). After infection, the larvae were treated with sterile PBS, FLC (160 μg/mL), LZD (200 μg/mL), and LZD (200 μg/mL) plus FLC (160 μg/mL), respectively and incubated. Three larvae from each group were randomly selected daily and were homogenized to determine *C. albicans* burden by inoculating dilutions of the homogenized larvae onto YPD solid medium plates as described above. For clarity, the fungal burden of the LZD group is not shown because the data were similar to that of the control group. The data came from the means of three independent experiments, and Student’s *t*-test was performed. Compared with the FLC-treated group, ^∗∗^*P* < 0.01.

Fungal burden analysis was conducted to detect the interactions of LZD with FLC on the fungal burden of *G. mellonella larvae* infected with CA10. Based on the data in Figure 3, a gradual increased fungal burden was observed in all groups, and there was obvious similarity in the fungal burden between the drug monotherapy groups and the control group. Encouragingly, the fungal burden of the LZD plus FLC group was significantly lower than that of the control group and drug monotherapy groups (*P* < 0.01), indicating that LZD plus FLC significantly decreased the fungal burden of the infected larvae.

#### Histological Study

**FIGURE 4:**
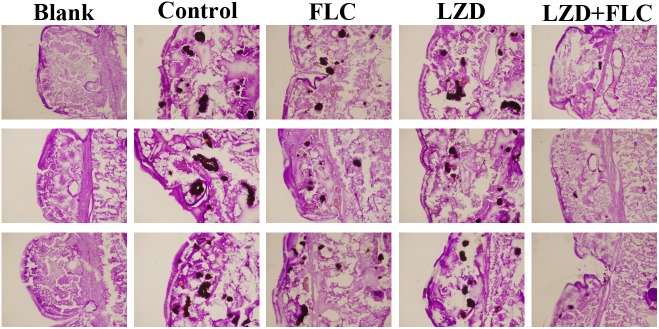
Histopathology of infected *G. mellonella* larvae treated with different drugs. CA10 (5 × 10^8^ CFU/mL) was used to infect the *G. mellonella* larvae (20 larvae for each group). After infection, the larvae were treated with sterile PBS, FLC (160 μg/mL), LZD (200 μg/mL), and LZD (200 μg/mL) plus FLC (160 μg/mL), respectively. Two larvae from each group were randomly chosen after 2 days of incubation and cut into 20-μm sections. Sections were stained with PAS reagent and observed under the fluorescence microscope. The larvae of the blank groups were not treated with yeast and drugs. Tissue sections were observed using 4.2 × 10 magnifications, with a scale of 200 μm.

A histological study was performed to characterize the infected tissue of *G. mellonella* larvae after treatment with different drugs. As shown in Figure 4, the tissue in the blank group was integrated and dense, with uniform staining and no black mass. In other groups, the larva tissues infected by CA10 presented as black lumps after PAS staining, and the number and area of the black lumps were different in the different groups. More specifically, black lumps in the control group and the drug-monotherapy groups were numerous and large, whereas those in the LZD plus FLC group were obviously much fewer and smaller, suggesting that compared with the FLC monotherapy, LZD plus FLC could significantly reduce the tissue damage of *G. mellonella* larvae caused by the resistant *C. albicans*.

## Discussion

In recent years, the emergence of drug-resistant *C. albicans* has placed a strain on health care systems and highlighted the need for effective approaches to overcome the resistance of *C. albicans* to antifungals. To solve this problem, the search for new antifungal drugs or sensitizers of existing antifungal agents has received wide attention. Numerous studies on antifungals sensitizers have found that many antibacterials or their analogs could enhance the efficacy of antifungals against resistant *C. albicans* (Shrestha et al., 2015; Eldesouky et al., 2018). LZD is a commonly used drug against gram-positive bacteria in clinical settings, and it also displays inhibitory effects against *Mycobacterium tuberculosis* (Lee et al., 2015), *Leishmania tropica* (Limoncu et al., 2013) as well as *P. insidiosum* (Loreto et al., 2014), and a synergistic effect against *C. neoformans* when combined with amphotericin B (Rossato et al., 2015). However, there is no report on the antifungal activities of LZD against *C. albicans* or the interactions of LZD with azoles. Inspired from the studies on the antifungal effects of LZD, we systematically evaluated the antifungal activities of LZD and the interactions of LZD and azoles against *C. albicans in vitro* and *in vivo*. The results show that LZD exerts a very weak anticandidal effect with MICs > 512 μg/mL but induces strong synergistic effects against *C. albicans* when used in combination with azoles. We were pleasantly surprised to find that LZD in combination with azoles induces synergistic effects not only against some susceptible *C. albicans* isolates, especially that of the combination of LZD and VRC, but also against all tested resistant *C. albicans* isolates with FICI values in the range of 0.03–0.19. The mechanisms of the synergistic effects need to be further explored.

Biofilm-related infections are difficult to treat in clinical settings because they tend to be chronic and easily recur (Desai et al., 2014). Biofilm formation has been demonstrated to be related to the drug resistance of *C. albicans* (Mathe and Van Dijck, 2013). The resistance induced by biofilm formation has seriously hampered the clinical treatments of candidiasis. In this study, we found LZD significantly synergized with FLC against *C. albicans* biofilms preformed for ≤ 12 h, and the sMIC_80_ of FLC decreased from >1024 to 1–4 μg/ml. With the preformed time extension, the biofilms were more mature, and the synergism was weaker. For biofilms preformed over 24 h, indifference was observed, indicating that LZD plus azoles might be a potential drug combination for the prevention or early treatment of biofilm-related diseases.

The *G. mellonella* larva infection model is a type of insect infection model that can be used to study the virulence of the pathogen, efficacy and toxicity of drugs (Lionakis, 2011; Champion et al., 2016; Tsai et al., 2016). Compared with the common mammal hosts, the *G. mellonella* larvae infection model can provide a rapid evaluation of the *in vivo* efficacy and toxicity of agents and the virulence of the pathogen with significant ethical and economic advantages (McMillan et al., 2015; Binder et al., 2016). In this study, we used this model to evaluate the *in vivo* combined effects of LZD and azoles and determined the survival rate of the larvae for a primary evaluation. The survival rates of the larvae treated with LZD + azoles were more than three- to fourfold higher than that of the control group, and twofold higher than that of azole monotherapy groups, demonstrating that LZD could significantly enhance the efficacy of azoles against resistant *C. albicans in vivo* (*P* < 0.01). Furthermore, the fungal burden analysis and histopathological study proved that LZD combined with azoles protected *G. mellonella* larvae against experimental candidiasis. Although there was a gradual increased fungal burden in all groups, a much lower fungal burden was observed in the LZD + FLC group than in the other groups, showing more efficacious effects for GM + FLC therapy than FLC monotherapy in clearing *C. albicans* from the larvae. The histopathological study of the larvae was performed to further study the *in vivo* interaction of drug combination. Compared with the blank group, the tissue of the larvae infected by the drug-resistant *C. albicans* was fragmentary, and the infected area showed black lumps after staining, indicating that the resistant *C. albicans* caused serious tissue damage to the tissues of the larvae. In addition, compared with the control group and the drug-monotherapy groups, fewer and smaller black lumps were observed in the LZD + FLC group. These findings showed that at the experimental concentration, LZD plus FLC could obviously weaken the damage of CA10 to the larvae. Taken together, LZD can significantly enhance the efficacy of FLC *in vivo*, which was in accordance with the *in vitro* results.

Linezolid is an oxazolidinone antibiotic that inhibits initiation of bacterial protein synthesis through a unique mechanism of action by binding to the 23S rRNA of the 50S ribosomal subunit on the bacterial ribosome, preventing the formation of functional 70S initiation complexes (Zahedi Bialvaei et al., 2017). Gemmell and Ford (2002) found that LZD was also effective in preventing the virulence factor synthesis of streptococcal and staphylococcal bacteria, such as hemolysins, coagulase and protein A. In our study, we also studied the impact of this drug combination on the efflux pump, one of the most common fungal resistant mechanism and fungal virulence, but no relation was found between the synergism and efflux pump activity or the virulence factor phospholipase activity. In a search of the literature, we found some studies on the mitochondrial toxicity of LZD, which demonstrated that LZD inhibited mitochondrial protein synthesis and interfered with the induction of stress-response mitochondrial chaperones (De Vriese et al., 2006; Santini et al., 2017). This finding provides us new ideas for further studies on the relation between mitochondrial function and synergism.

In conclusion, this paper provides an advance over our recent studies and in the field by first finding that LZD combined with azoles induced synergistic effects against *C. albicans* and protected *G. mellonella* larvae against experimental candidiasis. The findings in this paper suggest that the combination of LZD with azoles may hopefully be a therapeutic approach for resistant *C. albicans* infections, especially in areas with a high azole resistance rate of *C. albicans* or with an increasing trend of resistance to azoles. This study, together with the studies on the antifungal activities of LZD, will provide new potential approaches for the treatment of fungal infections caused by some pathogens, such as *C. albicans*, *P. insidiosum*, and *C. neoformans*. Future experiments will be conducted to investigate the relationship between mitochondrial function and synergism.

## Author Contributions

ML and SS conceived and designed the experiments. ML performed the experiments. ML, XY, CY, YG, LY, and SS analyzed the data and contributed to reagents, materials and analysis tools. ML and SS wrote the paper. All authors approved the manuscript for publication.

## Conflict of Interest Statement

The authors declare that the research was conducted in the absence of any commercial or financial relationships that could be construed as a potential conflict of interest.

## Abbreviations

CA: *C. albicans*
FLC: Fluconazole
*G. mellonella*: *Galleria mellonella*
ITZ: Itraconazole
LZD: Linezolid
VRC: Voriconazole.
