# Supplementary material for: Linezolid in Combination With Azoles Induced Synergistic Effects Against Candida albicans and Protected Galleria mellonella Against Experimental Candidiasis
Source: Front Microbiol. 2019 Jan 31;9:3142. doi: 10.3389/fmicb.2018.03142 (PMC6365414; doi:10.3389/fmicb.2018.03142)
Supplement: Supplementary file 1 [file Table_1.DOC]

Biofilms Production

Determination of biofilm production was determined using the method described by Shin et al. with some modifications. Briefly, a suspension was prepared and adjusted to 2.5 x 10^7^ CFU/mL. Then, 20 µL of this suspension was placed in the well of 96-well microtitration plates, and 180 µL of SDB supplemented with glucose (final concentration 8%) was added. Plates were then incubated at 35°C for 24 h without agitation. After incubation, the plate was washed twice with sterile phosphate-buffered saline (PBS), and the optical density (OD) was determined at 492 nm in a microtitration plate reader. The OD values obtained were used to calculate percent transmittance (%T) values. The %T value for each test sample was subtracted from the %T value of the blank to obtain the %Tbloc. The interpretation scale used was: negative (%T < 5), + (%T 5-20), + + (%T 20-35), + + + (%T 35-50), + + + + (%T > 50).

Table Biofilms Production of all isolates used in this study.

| **Isolates** | **Mean % T value ± SD** | **Biofilms Production** |
| --- | --- | --- |
| CA4 | 72.87 ± 4.37 | **+ + + +** |
| CA8 | 79.07 ± 3.56 | **+ + + +** |
| CA14 | 26.76 ± 4.17 | **+ + + +** |
| CA17 | 5.51 ± 3.09 | **+** |
| CA19 | 74.31 ± 1.39 | **+ + + +** |
| CA23 | 78.97 ± 3.67 | **+ + + +** |
| CA10 | 72.72 ± 0.91 | **+ + + +** |
| CA16 | 77.01 ± 4.05 | **+ + + +** |
| CA103 | 74.72 ± 7.84 | **+ + + +** |
| CA137 | 74.74 ± 5.55 | **+ + + +** |
| CA632 | 71.53 ± 6.06 | **+ + + +** |
| CA20003 | 30.33 ± 3.22 | **+ +** |
